# 2D Ruddlesden–Popper Perovskites with a Thick Octahedral Layer (*n* = 7) as a Robust Alternative for Energy-Related Application

**DOI:** 10.1021/acsphyschemau.6c00021

**Published:** 2026-05-08

**Authors:** Aryane Tofanello, André L. M. Freitas, Fabio Abud, Leonardo Quintero, Ulisses F. Kaneko, Ricardo D. Reis, Jose A. Souza

**Affiliations:** † Center for Natural and Human Sciences (CCNH), Federal University of ABC (UFABC), Santo André, São Paulo 09210-580, Brazil; ‡ Brazilian Synchrotron Light Laboratory (LNLS), Brazilian Center for Research in Energy and Materials (CNPEM), Campinas, São Paulo 13083-100, Brazil; § São Paulo State University - UNESP, Institute of Geosciences and Exact Sciences (IGCE), Physics Department, Rio Claro, São Paulo 13506-900, Brazil

**Keywords:** two-dimensional hybrid perovskite, quasi-3D halide perovskites, environmental stability, pressure effect, photoconductivity, energy-related application

## Abstract

Halide perovskites have led to outstanding optoelectronic performance, even though their operational stability, especially for 3D MAPbI_3_, persists as a critical obstacle. Here, we synthesize a Ruddlesden–Popper iodide series (*n* = 1, 2, and 7) using a butylammonium spacer and directly compare the thick-layer member (*n* = 7; hereafter termed *quasi*-3D) with 3D MAPbI_3_. Structural and morphological analyses confirm a progressive evolution toward 3D-like character with increasing *n* (octahedral sheets), while steady-state optical measurements show that the *n* = 7 phase exhibits an emission energy close to MAPbI_3_. Crucially, *in situ* diamond-anvil-cell photoluminescence under hydrostatic pressure reveals markedly different flexibility: MAPbI_3_ undergoes strong PL quenching at lower pressure and shows incomplete spectral recovery upon decompression, whereas the *quasi*-3D phase sustains emission to substantially higher pressures and displays reversible PL restoration after pressure release. Complementary surface measurements show enhanced hydrophobicity for the *quasi*-3D sample, and electrical conductivity demonstrates a comparable light-induced photoconductivity response to that of MAPbI_3_. Our results suggest that thick-layer (*n* = 7) Ruddlesden–Popper perovskites are a promising route to improve chemical and mechanical stability while preserving 3D-like optoelectronic functionality for energy-related devices.

## Graphical abstract

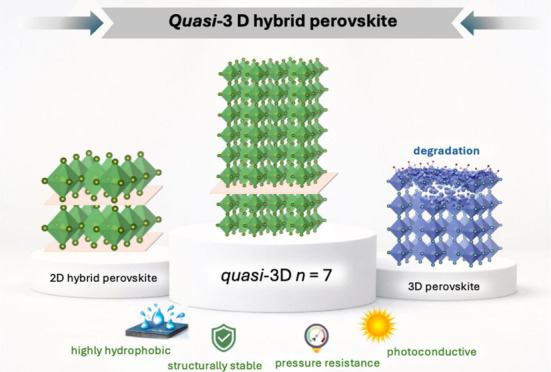

## Introduction

Hybrid lead halide perovskites have emerged as a versatile class of semiconductors for optoelectronic and energy-related applications, including photovoltaics, light-emitting diodes, and photodetectors, owing to their desirable solution-processing method and correlated physical properties.^1^ Among them, three-dimensional (3D) perovskites such as MAPbI_3_ exhibit outstanding optoelectronic performance; however, their practical implementation remains hindered by limited operational stability.^2,3^ Sensitivity to moisture, light, heat, and mechanical stress, together with ion migration and defect-assisted degradation, continues to represent a major obstacle to long-term device reliability.^4,5^ Dimensionality engineering via layered Ruddlesden–Popper (RP) perovskites has proven to be an effective strategy to mitigate these limitations. In RP structures, bulky organic spacer cations separate inorganic Pb–I octahedral slabs, improving moisture resistance and structural robustness.

Indeed, these two-dimensional (2D) counterparts of halide perovskites have emerged as a more versatile family of semiconducting materials concerning stability, crystal structure, synthetic versatility, stronger quantum confinement, greater wavelength tunability, and facility for device implementation.^4–7^ The general formula is given by (OS)_2_(MA)_n–1_Pb_n_X_3n+1_, where MA = CH_3_NH_3_^+^, X = Cl^–^, Br^–^, I^–^, and OS is an organic spacer such as RNH_3_.^7,8^ The Pb_n_X_3n+1_ arrangement defines the inorganic layer and the spacer cations noncovalently hold the octahedral slabs. The thickness of the inorganic layer depends on the parameter *n*, which represents the number of corner-shared PbX_6_ octahedra layers sandwiched between an insulating layer of amines. Modulating the *n* value allows considerable electronic and structural tunability, influencing key physical parameters such as band gap energy, exciton binding energy, carrier mobility, optical absorption, and photoluminescence. Notably, the growth of this 2D phase can be easily obtained through a one-step chemical method or by water-induced recrystallization.^9,10^ For example, the rational choice of organic spacers can induce an additional spin degree of freedom in these 2D systems, while chiral ligands enable tuning of the broadband emission arising from self-trapped excitons.^11,12^ Additionally, the electronic structure and dynamics of excited charge carriers can be effectively manipulated in the 2D systems by engineering the crystal structure giving rise to different excitonic emissions and unique optoelectronic phenomena.^12–17^ Their organic–inorganic layered structure (multiple quantum well structure) creates higher migration barriers, suppressing vacancy-mediated motion and lowering the density of mobile ionic defects.^18,19^ However, low-*n* RP phases exhibit strong quantum and dielectric confinement, resulting in large exciton binding energies and hindered charge transport across layers, which can severely limit device performance. This inherent compromise between stability and optoelectronic functionality has motivated the search for an optimal intermediate dimensional regime.

*Quasi*-3D RP perovskites, characterized by thick inorganic slabs (large *n*) and a reduced fraction of organic spacers, have recently emerged as promising candidates to reconcile these competing requirements. As *n* increases, dielectric confinement weakens and the electronic structure approaches that of 3D perovskites, enabling increasingly free-carrier-like behavior while potentially retaining some stability benefits imparted by the organic spacers. Despite this promise, the physical properties of thick-layer RP perovskites remain comparatively underexplored, particularly under external perturbations that directly probe lattice flexibility, defect tolerance, and reversibility. Hydrostatic pressure provides a powerful and chemically clean tool to investigate the coupling between lattice distortions and optoelectronic response in halide perovskites.^20,21^ By continuously tuning interatomic distances without introducing chemical disorder, pressure experiments directly probe mechanical resilience and structural stability. While 3D halide perovskites are known to undergo early photoluminescence quenching, amorphization, and irreversible degradation under pressure, a systematic comparison with quasi-3D RP systems is still missing. In this work, we address this gap by synthesizing a series of butylammonium-based Ruddlesden–Popper iodide perovskites with *n* = 1, 2, and 7, together with their 3D counterpart MAPbI_3_, and by performing a direct, comparative study of their structural, optical, surface, and transport properties. Focusing on the thick-layer *n* = 7 compound, we combine steady-state spectroscopy, surface wettability, photoconductivity measurements, and *in situ* diamond-anvil-cell photoluminescence under hydrostatic pressure. Our study establishes the quasi-3D (*n* = 7) RP perovskite as a distinct regime that preserves 3D-like photophysics and charge transport while exhibiting enhanced mechanical and moisture stability, thereby defining its scope and relevance as a robust platform for energy-related optoelectronic devices.

## Materials and Methods

### Chemicals and Materials

Lead­(II) oxide (≥99%), butylamine (≥99%), hydriodic acid (57 wt % in H_2_O, 99.95%) and hypophosphorous acid solution (50 wt % in H_2_O) were purchased from Sigma-Aldrich and all chemicals were used without further purification. For the synthesis of methylammonium chloride (MACl), aqueous methylamine (MA, 40 wt % in H_2_O) was dropped into hydrochloric acid (HCl, 32 wt % in H_2_O) solution with a molar ratio of 1:1 at 0 °C accompanied by stirring for 2 h. The mixed solution was evaporated at 60 °C, washed three times after, and then dried at 70 °C to obtain the MACl powder.

### Perovskite Synthesis

The 2D structures were synthesized with the model molecule butylammonium as a spacer and methylammonium, while their three-dimensional forms contained only methylammonium as the central cation. Layered halide perovskite platelets and MAPbI_3_ were synthesized through a facile solution-processed method reported by Soe et al., with slight modifications.^22^

### MAPbI_3_ (n = ∞)

0.111 g of PbO powder was dissolved in a mixture of 0.5 mL of HI solution and 85 μL of aqueous H_3_PO_2_.The system was heated at 140 °C under constant magnetic stirring for 20 min, forming a bright yellow solution. In sequence, 0.033 g of solid MACl was added to the hot precursor solution, immediately causing precipitation of a black powder. The solution was stirred for 10 min and rinsed three times with toluene and dried at 60 °C.

### (BA)_2_PbI_4_ (*n* = 1)

111.6 mg of PbO powder was dissolved in a mixture of 0.5 mL of HI solution and 85 μL of aqueous H_3_PO_2_.The system was heating at 140 °C under constant magnetic stirring for 20 min, forming a bright yellow solution. In a separate glass flask, 5 μL of butylamine was neutralized with 1 mL of HI in an ice bath. The chilled BA-I was added to the lead precursor solution, which subsequently dissolves upon heating the solution to boiling. The stirring was discontinued, and the solution was allowed to cool to room temperature during which time orange crystals begin to precipitate. The crystals were rinsed three times with toluene or hexane and dried at 60 °C.

### (BA)_2_(MA)_2_Pb_3_I_10_ (*n* = 2)

111.6 mg of PbO powder was dissolved in a mixture of 0.5 mL of HI solution and 85 μL of aqueous H_3_PO_2_.The system was heating at 140 °C under constant magnetic stirring for 20 min, forming a bright yellow solution. Next, 0.225 mg of MACl were incorporated to the hot precursor solution, immediately precipitating a black powder, which quickly dissolved under stirring to afford a clear and bright yellow solution. In a separate glass flask, 3.5 μL of butylamine was neutralized with 2 mL of HI in an ice bath. The chilled BA-I was added to the lead precursor solution, which subsequently dissolves under heating the solution to boiling. The stirring was discontinued, and the solution is allowed to cool to room temperature during which time red crystals begin to precipitate. The crystals were rinsed three times with toluene or hexane and dried at 60 °C.

### BA_2_MA_6_Pb_7_I_22_ (*n* = 7)

111.6 mg of PbO powder was dissolved in a mixture of 0.5 mL of HI solution and 85 μL of aqueous H_3_PO_2_.The system was heating at 140 °C under constant magnetic stirring for 20 min, forming a bright yellow solution. Next, 0.028 mg of MACl were incorporated to the hot precursor solution, immediately precipitating a black powder, which quickly dissolved under stirring to afford a clear and bright yellow solution. In a separate vial, BA was neutralized with HI (1:1) in an ice bath, the excess acid was used to ensure that the amine group was completely protonated. Then, 8.3 μL of chilled BAI solution was dropped to the previous hot solution, initially produces a black precipitate, which subsequently dissolved under heating the solution to boiling. In this step, the stirring was discontinued, more 8.3 μL of chilled BAI solution was added and small dark mulberry color plate-like crystals precipitated immediately upon the insertion of the protonated cation. The layered halide perovskite platelets were rinsed three times with toluene and dried at 60 °C.

### Characterization

X-ray diffraction (XRD) measurements were carried out with a Bruker D8 Discover X-ray diffractometer operating at 40 kV and 40 mA with a Cu Kα anode (λ = 0.1542 nm). Scanning electron microscope (SEM) images were acquired at an accelerating voltage of 10 kV using JSM-6010LA (JEOL). Photoluminescence measurements were acquired using a FluoroMax-4P TCSPC spectrofluorometer produced by Horiba. The excitation source in this system was a 150-W ozone-free xenon lamp. The absorbance spectroscopy was carried out on Thermo Scientific Evolution Array UV–visible Spectrophotometer (South Korea). The samples were placed on a glass substrate and the spectra were collected from 400 to 850 nm. Contact angle measurements with water were conducted on DSA 100 contact angle measuring device (KRUSS GmbH). The droplet volume was 2 μL. X-ray photoelectron spectroscopy (XPS) measurements were performed on K-alpha+ ThermoFisher Scientific. The XPS data were fitted with Gaussian–Lorentzian functions with a Shirley type background. High-pressure XRD and high-pressure photoluminescence were conducted at the Extreme Methods of Analysis (EMA) beamline of the Brazilian Synchrotron Light Laboratory.^23^ In both experiments, high pressure was generated using a diamond anvil cell (DAC) equipped with 600 μm diamond anvils. A stainless-steel gasket with a 200-μm hole was used, and the sample, along with a ruby ball, was loaded into it. Neon served as the pressure medium. Pressure was controlled through a gas-membrane mechanism integrated with the DAC and calibrated in situ by measuring ruby ball fluorescence. Photoluminescence (PL) spectra as a function of pressure were obtained from an optical system in a confocal geometry, which allowed for microscopic imaging of the sample placed in the gasket hole inside the DAC. A 405 nm laser was employed as the excitation source, while the signal was collected by a Princeton HRS-300 spectrometer equipped with a back-illuminated PIXIS-BR100-excelon CCD camera.

## Results and Discussion

As described above, we have synthesized 2D Ruddlesden–Popper BA_2_(CH_3_NH_3_)_n‑1_Pb_n_I_3n+1_ samples by using *n*-butylamine (BA) molecules as a spacer with *n* = 1 and 2. Additionally, we have also synthesized a 2D sample with increased number of octahedral sheets *n* = 7, which is close to a 3D system. We have also produced its 3D counterpart MAPbI_3_ (MA= CH_3_NH_3_) to study the optical and structural evolution comparatively. An interesting way to illustrate the structure of each 2D inorganic layer is by slicing the 3D structure (*n* = ∞) along the (110) plane resulting in partial *n* = (2, 3, 4, ...) or completely (*n* = 1). X-ray powder diffraction was measured for all samples as shown in Figure S1. Interestingly, as one introduces a molecule spacer into 3D structure, additional low-angle Bragg reflections are observed due to larger interplane distance. For instance, the *n* = 1 layered structure exhibited a single reflection below ∼ 2θ = 14°. The *n* = 2 analogue showed two reflections (Figure S1 (red curve)) at the same range, and so on for members of the (BA)_2_(MA)_n–1_Pb_n_I_3n+1_ series. As expected, the 2D crystals with *n = 7* displayed several reflections before 2θ = 14°. The 2D structures crystallize in an orthorhombic structure while the 3D counterpart in a tetragonal space group. All the lattice parameters and unit cell volume are organized in the Table S1 of Supporting Information. The lattice parameters were refined using the *CellCal* software based on the CIF data from previous studies.^7,22,24^ Most important for 2D structured systems is the layer-stacking direction. For this reason and for a better comparison, we have aligned the crystal axes for the three samples so that the layer-stacking direction points along the *b*-axis while the *a*- and *c*-axis define the two in-plane directions of the perovskite 2D layer, see Table S1.

In order to morphologically characterize the perovskite structures, scanning electron microscopy and optical images are displayed in Figure 1. The RP layered perovskite compounds usually precipitate in the form of colored rectangular-shaped plates with the spectral range spanning from yellow to black, depending on the thickness of the octahedral layer, or *n* value. It can be clearly observed that each *n* corresponds to its respective characteristic color: yellow, red, plum, and black, for *n* = 1, 2, 7, and ∞, respectively, as reported in previous works.^7,13,25^ Furthermore, as the value of *n* increases, the crystals tend to become thicker while exhibiting relatively low levels of translucency. It is known that this type of structure normally presents itself as thin plates for *n* between 1 and 4.^26,27^ As can be seen in Figure 1, for both *n* = 1 and *n* = 2, the perovskite crystals grow in the form of microplates, in which the lateral dimensions are close to 50 ± 8 μm and 62 ± 12 μm and the thickness is approximately 1.3 ± 0.4 μm and 2.1 ± 0.3 μm, respectively. For *n* = 7, there is a presence of similar plate-like structures that tend toward a dark color, such as plum (Figure 1c), while the 3D perovskite is presented in the form of black cubes (Figure 1d). The *quasi-*3D microplates exhibit lateral dimensions close to 73 ± 9 μm and a thickness of 3.3 ± 0.4 μm. A deeper look can reveal a slight tendency for rounded edges in the particles, as well as an increase in thickness, which would lead to the formation of three-dimensional cuboids when extrapolating the value of *n* (Figure 1d). Therefore, as the *n* value increases, the crystals tend to become thicker likely related to the decrease of van der Waals layers present between spacers.

**Figure 1:**
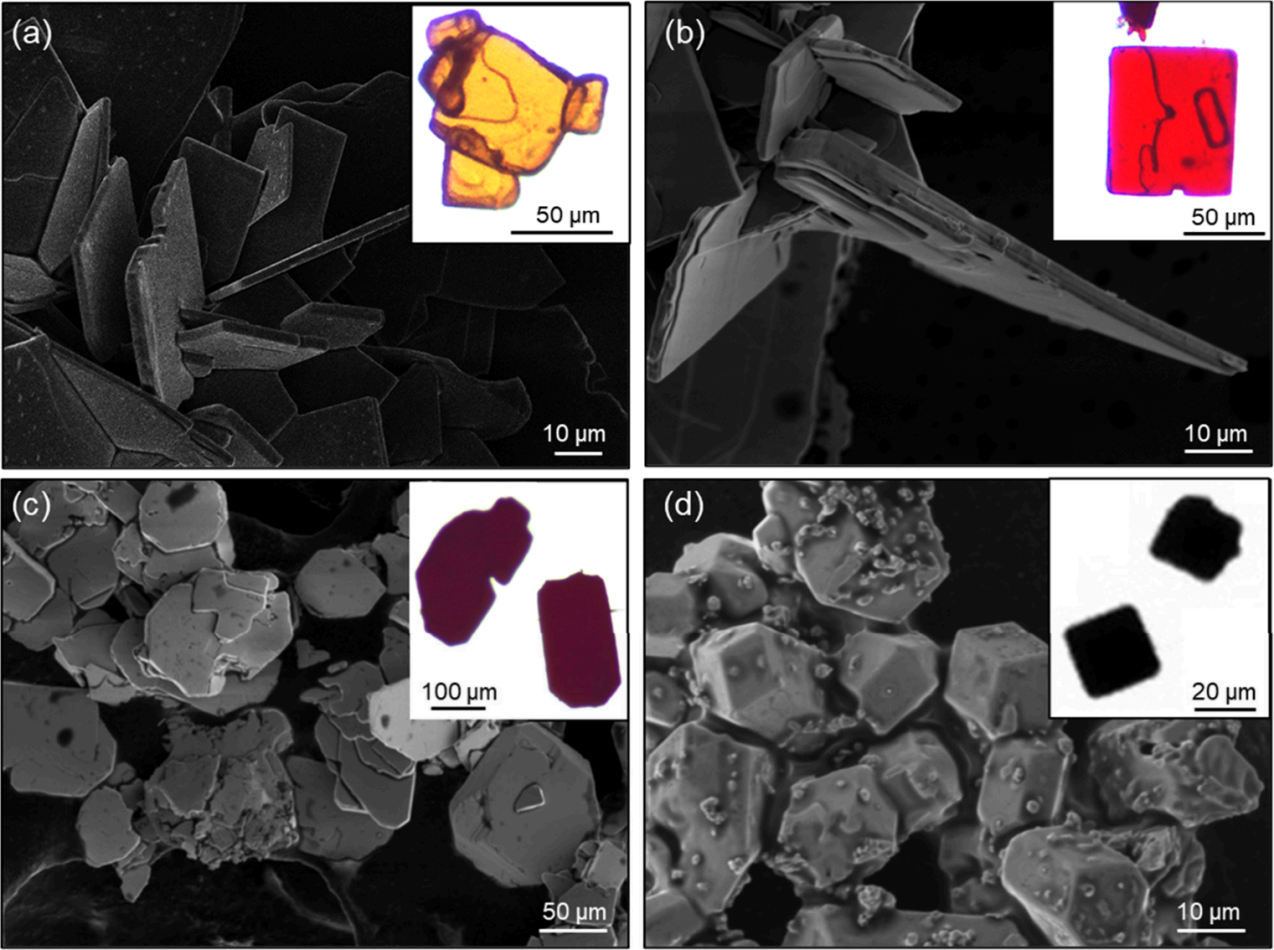
SEM and optical images of 2D perovskite with plate-like crystal morphology for *n* = 1 (a), *n* = 2 (b), *n* = 7 (c) and cuboid shape for *n* = ∞ (d).

Next, the room-temperature optical properties of the as-grown perovskite were examined by UV–vis absorption and steady-state photoluminescence (PL) spectroscopy. The spectra exhibit clear trends associated with excitonic behavior and quantum-well confinement, which depend strongly on the number of inorganic layers in the Ruddlesden–Popper structure. Figure S2 displays the absorption profiles of the as-synthesized perovskites crystals. The cutoff absorption edges of the *n* = 1 and *n* = 2 phases appear at approximately 530 and 580 nm, corresponding to optical band gaps of 2.33 and 2.13 eV, respectively, in agreement with the expected strong excitonic character of low-*n* layered perovskites. As the layer thickness increases, the absorption onset experiences a systematic red-shift, reflecting the progressive reduction in quantum confinement. These confinement effects are further confirmed by the PL spectra shown in Figure 2. The *n* = 1 and *n* = 2 samples display intense emission peaks at 508 nm (2.44 eV) and 582 nm (2.13 eV).^28^ Figure 2 also shows a prominent emission peak at 746 nm, which can be assigned to 2D perovskite with *n* = 7.^29–31^ The photoluminescence curve for 3D MAPbI_3_, corresponding to *n* = ∞ phase, shows an emission peak at 770 nm. Notably, the emission peak of the *n = 7* sample lies very close to the 3D sample, even though the layer thickness is around 102.98 Å, i.e., with still quantum confinement, as observed for the Bohr radius of halide perovskites.^32^ The Bohr radius serves as a limit to separate bulk and quantum confinement properties.

**Figure 2:**
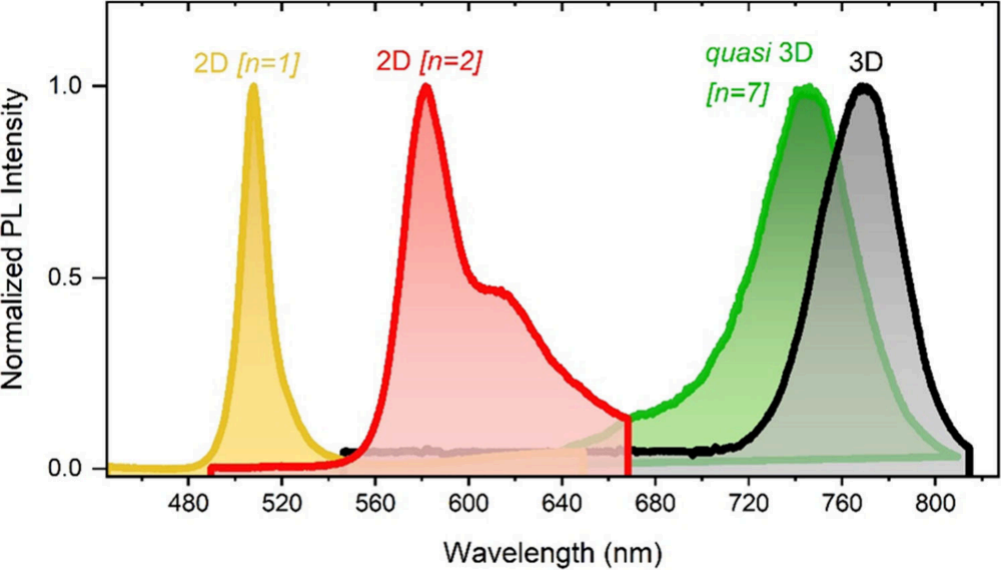
Normalized PL emission under 401 nm excitation spectra of 2D perovskite with *n* = 1 (yellow curve), *n* = 2 (red curve), *n* = 7 - *quasi* 3D (green curve) and 3D perovskite (black curve) at room temperature.

In semiconductors, photogeneration, exciton dissociation, and charge-carrier transport are crucial for energy-related devices. The enhanced quantum and dielectric confinement observed in 2D layered perovskites contributes to a significantly larger excitonic binding energy compared to their 3D counterparts. When *n* is small, typically *n* < 3, charge carrier recombination is significant due to strong electron–hole binding energy. In low-*n* 2D perovskites, the combination of a high exciton binding energy and the presence of organic spacers may represent a significant transport barrier across adjacent 2D sheets, which is unfavorable for charge separation in solar cells. However, as *n* increases, the binding energy decreases, which facilitates the charge separation. For infinitely thick perovskite layers or 3D perovskites, free electrons and holes are the main photocarriers observed in the optical excitation and recombination kinetics. In contrast, however, predicting the implications of these effects on optical properties of 2D hybrid perovskites remains nontrivial due to the differences in the chemical composition of 3D and 2D perovskites. Given that *quasi*-3D crystals represent the optimization limit of lamellar perovskites, we will focus our investigation on the *quasi*-3D and 3D samples to comparatively explore their physical properties.

To further understand the difference in the chemical states and environments of the ions, we have performed high-resolution XPS. The high-resolution XPS core-level I 3d doublets, N 1s, C 1s, and Pb 4f doublets for both samples are depicted in Figure 3. In the Pb 4f spectrum of 3D MAPbI_3_, there are two main peaks located at 138.78 (Pb 4f_7/2_) and 143.58 eV (Pb 4f_5/2_), stemming from Pb^2+^ cations, corresponding to the Pb–I binding energy in the perovskite (Figure 3a). Compared with bulk perovskites, the Pb 4f peaks in *quasi*-3D crystals displayed a shift to a higher binding energy. The 2D spectrum showed two main peaks at 143.78 eV (Pb 4f_5/2_) and 138.78 eV (Pb 4f_7/2_) (Figure 3a). Furthermore, two minor peaks located at 144.18 and 139.38 eV can be attributed to the lead microenvironment with organic molecules.^33^ These observable differences in binding energies can be attributed to slightly different chemical environments of the Pb–I covalent bonds in the absence of butylamine molecule. Such an observation indicates that in the layered phase, there is a tendency for the elements to chemically rearrange themselves in order to adapt to the chemical structure of perovskites.^34^ Interestingly, for the *quasi*-3D crystals, a broadening followed by a shift in lead binding energy was observed. Changes in Pb binding energy reflect the more electron-rich environment (compared to 3D counterparts) around the Pb centers of two-dimensional perovskites, which is an inherent property resulting from their distinctly different chemical compositions: interface Pb with proximity to the butylamine content and lead situated deeper within the bulk of the octahedral layer (typical Pb^2+^ state). Similar significant changes in binding energies were also observed for the other constituent elements, which could be explained in a similar manner. In low-dimensional perovskites, the bonds and interactions between atoms are arranged to maintain a sphere of coordination with a −2 charge, contrasting with their 3D counterparts that exhibit a −1 charge. This arrangement significantly alters the electronic densities of the elements and is evident in the distinct ″status″ of the ligands.^35^ The spin–orbit split between the Pb doublet is 5 eV for both perovskite configurations, which is close to the reported values.^36^

**Figure 3:**
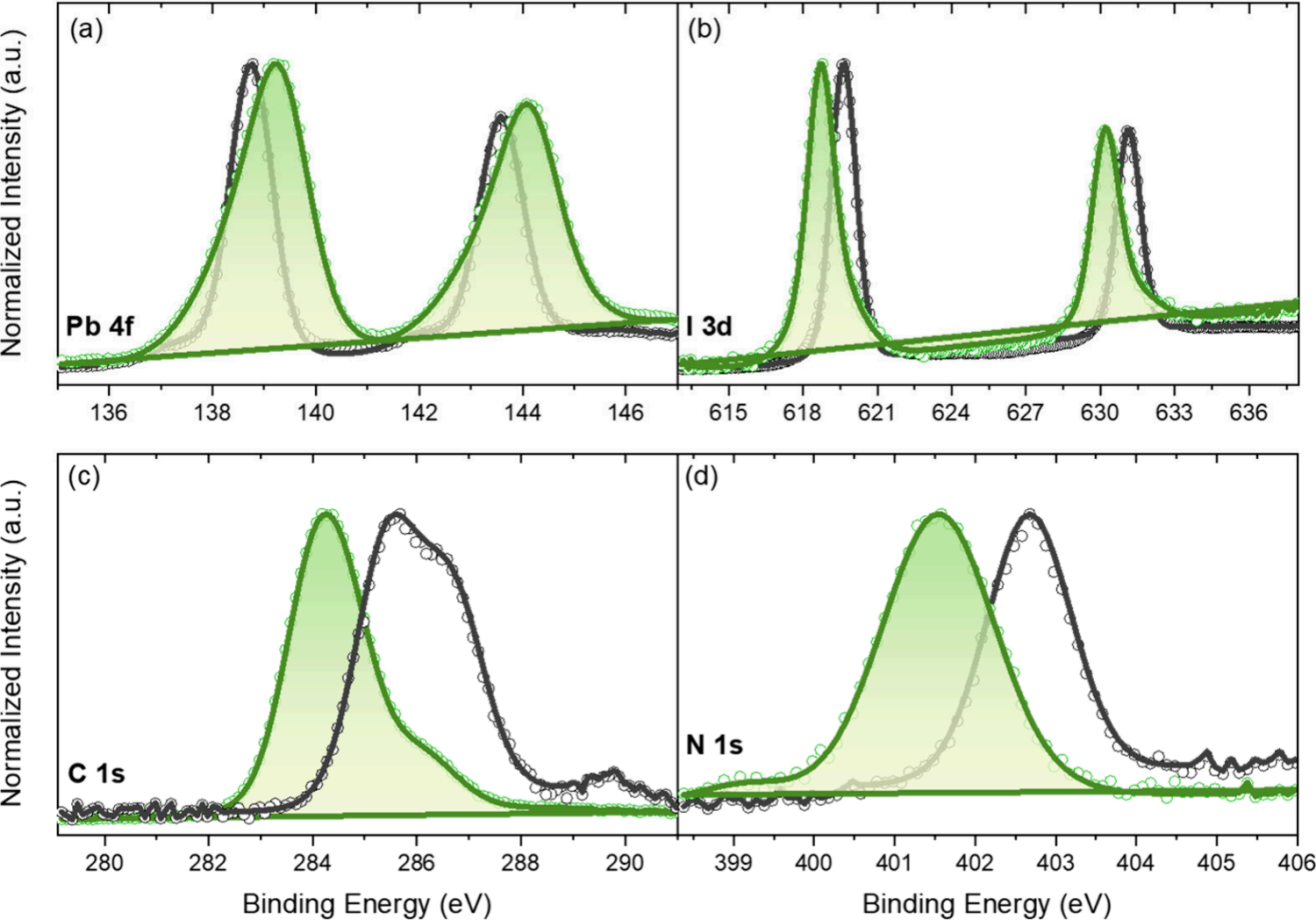
Core-level XPS spectra for 3D MAPbI_3_ (black line) and *quasi-3D* (green line) perovskites: (a) Pb 4f, (b) I 3d, (c) C 1s and (d) N 1s.

For the *quasi-*3D microplatelets, the high-resolution XPS I 3d doublet spectra (Figure 3b) exhibited peaks at 630.18 eV (I 3d_3/2_) and 618.68 eV (I 3d_5/2_) (which correspond to the I^2+^ charge state) and 630.8 and 618.98 eV (which correspond to the I^3–^ charge state).^33^ For the 3D sample, there was a slight shift toward a higher binding energy. In the case of layered perovskites, the coexistence of MA and BA cations can be distinguished from each other by the C 1s and N 1s spectra. Larger shifts in binding energy are observed in the 2D structure for these two elements, which can be justified by the additional presence of the spacer cation and its interface interactions. The C 1s spectra of the *quasi*-3D perovskite (Figure 3c) exhibited two prominent peaks at 283.90 eV, which corresponded to aliphatic hydrocarbon (C–H or C–C), and one at 284.78 eV, which corresponded to carbon atoms bonded with single nitrogen atoms (C–N). There was a shoulder in the C 1s spectra at 286.08 eV, which could be assigned as carbon-bound iodine. Notably, the deconvoluted N 1s spectra showed a contribution at about 401 eV, which could be split into two independent peaks as a consequence of the presence of two types of N in different chemical environments (Figure 3d). The N 1s spectra displayed a main peak located at 401.58 eV associated with the protonated nitrogen atoms (C–N in the amino group).^37^ The combined analysis between the peaks of the C 1s spectrum and N 1s spectrum can be considered evidence of C-NH_3_^+^ contribution in the butylamine cations. For the 3D MAPbI_3_ structure, the peaks around 285.58 eV (related to C 1s) and 402.7 eV (related to N 1s) belong to the MA^+^ cation and are in good agreement with previously reported data.^38^

Figure 4 shows the *in situ* hydrostatic pressure photoluminescence experiments for both *quasi-*3D and 3D samples. The photoluminescence emission peak of MAPbI_3_ is at 786 nm at ambient conditions (Figure 4c). Upon increasing pressure, a slight blue shift followed by an almost pressure independent behavior. Above 3 GPa, it was observed apparent total PL quenching. After the pressure release, besides a small blue shift in the peak, a strong suppression in PL intensity is observed, as shown in Figure 4d, related to induced disorder and lattice defects. The PL spectra evolution of the *quasi*-3D crystal differs markedly from that of the 3D sample. The *off DAC* PL emission exhibits an initial peak at 749 nm (Figure 4a). At 1 GPa, a high-energy emission peak at 675 nm appears, coexisting with that initial low-energy peak up to 2 GPa. Afterward, a considerable high-energy peak is observed up to 6 GPa attributed to low-*n* phases (coexistence), e.g., ∼580 nm for *n* = 2 phase; ∼620 nm for *n* = 3 phase; ∼650 nm for *n* = 4 phase; ∼ 680 nm for *n* = 5 phase, etc. The pressure-induced features are consistent with previously reported and likely reflect contributions from lower-*n* phases arising upon compression.^7,28,31^ The PL spectra after pressure release (Figure 4b), demonstrates the presence of minority phases - peaks around 580 and 620 nm. The emergence of higher-energy PL bands under compression in the n = 7 sample suggests the activation of emissive contributions compatible with lower-n Ruddlesden–Popper phases. At present, these features cannot be assigned uniquely to a single mechanism and may originate from pre-existing minority domains, pressure-induced local phase redistribution, or structurally distinct surface/interface regions that become optically active under compression. We therefore interpret these peaks as evidence of local heterogeneity revealed or enhanced by pressure, rather than a of complete bulk transformation. Importantly, the recovery of the main quasi-3D emission after decompression still demonstrates that the dominant n = 7 framework remains substantially more reversible than 3D MAPbI_3_.

**Figure 4:**
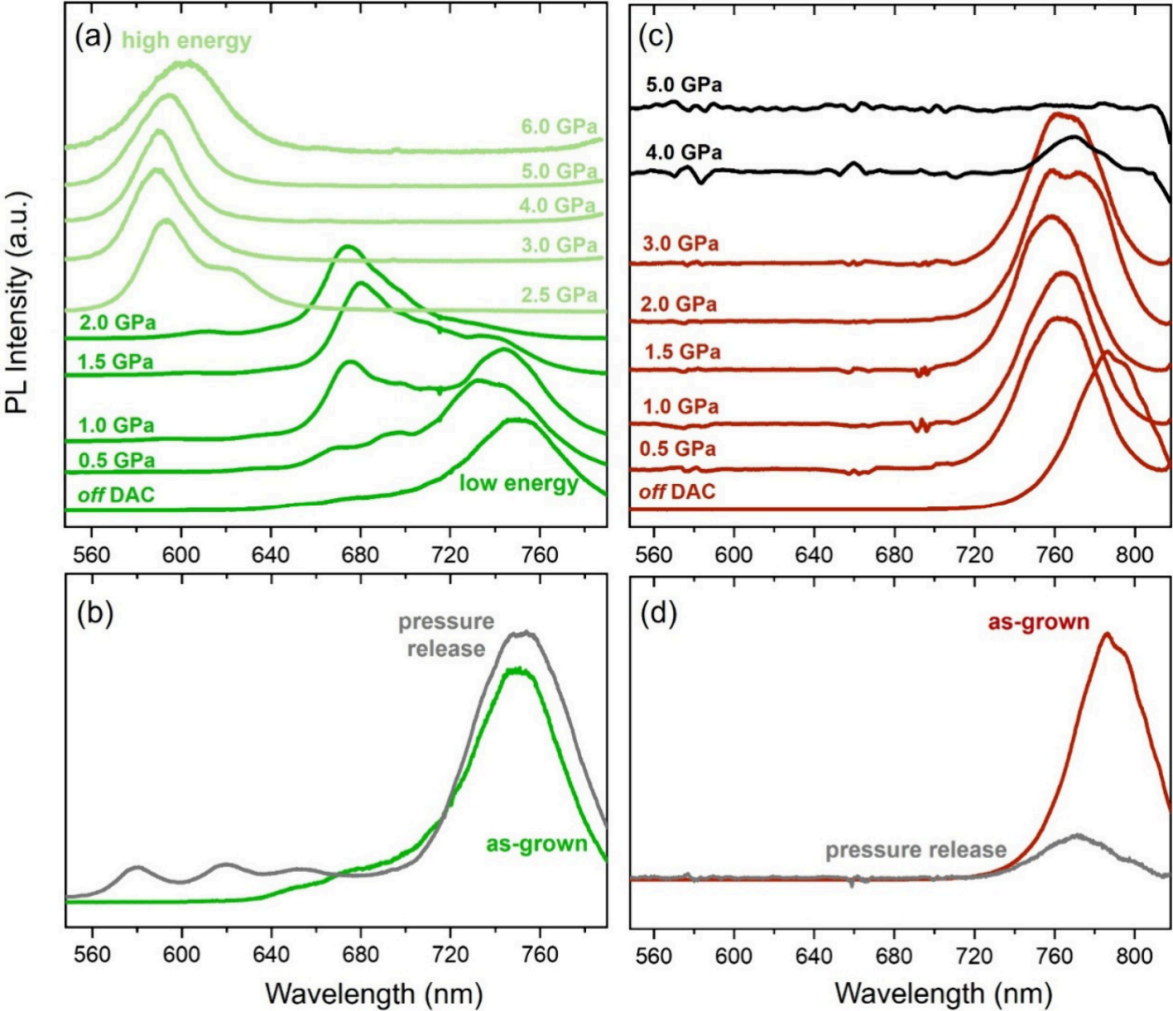
Photoluminescence as a function of some representative pressure for (a) *quasi-3D* crystals and (c) for 3D perovskite. PL comparison of the as-grown sample with after pressure annealing for *quasi-*3D crystals (b) and for 3D perovskite (d).

Remarkably, upon pressure release, the excitonic peak reappears, evidencing a reversible transformation of the emission in the *quasi*-3D system. Figure 4b compares the PL spectra of the as-grown *quasi*-3D sample and the pressure-released sample, clearly demonstrating this reversibility. The 3D sample exhibits pronounced PL quenching at 4 GPa, whereas the *quasi*-3D sample maintains persistent emission up to higher pressures. Following pressure annealing, the 3D sample displays a sharp and abrupt reduction in emission intensity, as shown in Figure 4d. In contrast, the *quasi*-3D sample presents an emission intensity higher than that of the pristine crystal (Figure 4b), indicating enhanced structural stability and a lower pressure-induced defect formation. Therefore, we observed that when subjected to mechanical stresses such as bending, stretching, or impact, 2D hybrid perovskites can recover their original structure without undergoing permanent amorphization, thereby preserving their optoelectronic properties. These pressure-dependent behaviors reveal a clear contrast in structural resilience between the *quasi*-3D and 3D lattices. The early amorphization and PL quenching observed for MAPbI_3_ indicate a mechanically weaker lattice and defect-assisted disorder. In contrast, the *quasi*-3D sample endures higher pressures and fully recovers its emission upon release, consistent with a more robust and defect-resistant lattice framework.

From now on, we examine the key material properties that determine the suitability of the *quasi*-3D perovskite for practical optoelectronic applications. Despite being relatively unexplored, we suggest that our 2D perovskites with large *n* represent a promising alternative for the design of optoelectronic devices. Incorporating them as heterostructures on a 3D surface (2*D*/3D) has also attracted attention due to their capability to increase photovoltaic performance with enhanced stability.^39–41^ The fundamental concern with hybrid halide perovskites lies in a delicate balance among structural stability, hydrophobicity, and charge conductivity. Although enhancing thermal stability and moisture resistance, the organic spacer also alters the charge transport dynamics, potentially reducing the electrical conductivity due to quantum wells limiting the device performance.^40,42,43^

First, we compare the wettability of both samples. The 3D crystals exhibited greater wettability compared to the *quasi*-3D perovskite in the initial configuration, as shown in Figure 5a–d, respectively, highlighting the hydrophilic behavior of the MAPbI_3_ structure. This suggests that the minority presence of the organic spacer compared to the octahedral layers in the *quasi-3D* perovskite was sufficient to protect the surface, as revealed by the observed increase in the contact angle. Subsequent measurement, conducted after a 1 min exposure to water, the *quasi*-3D sample (Figure 5b) maintained the hydrophobic trend indicating that the surface remains preserved over time. In contrast, the MAPbI_3_ sample was nearly completely wetted with water (Figure 5d).

**Figure 5:**
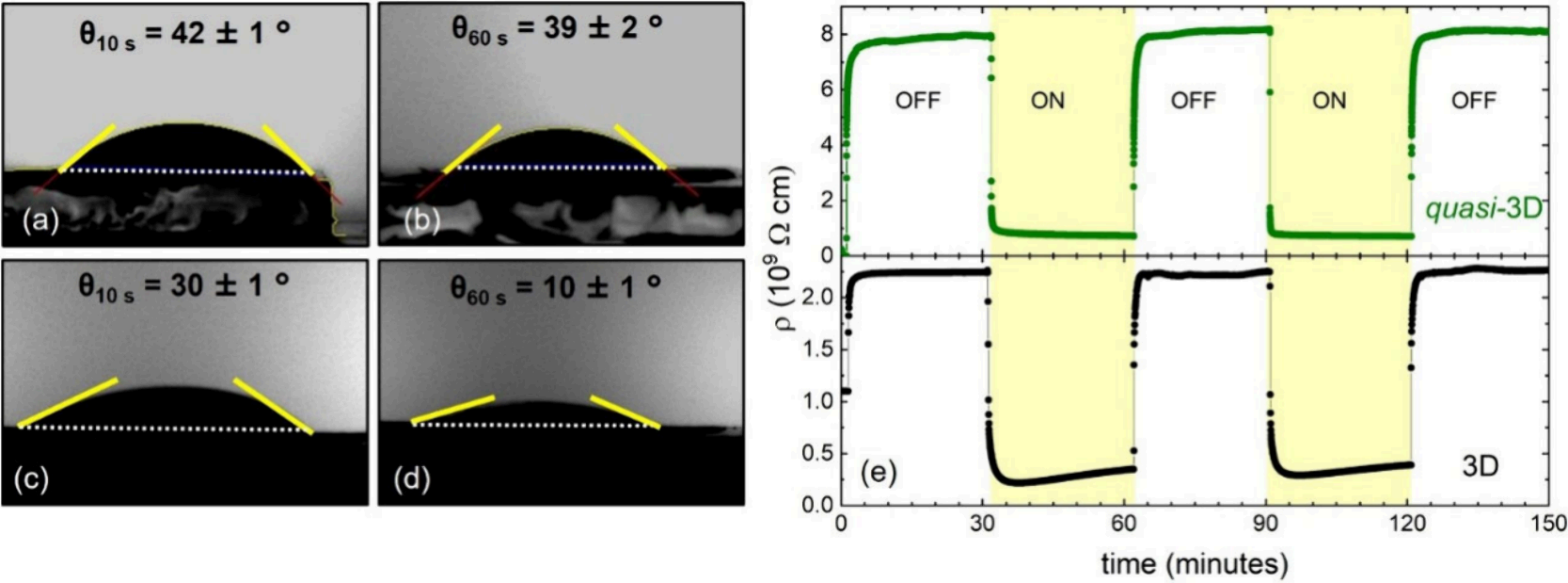
Contact angle showing wettability of both (a, b) *quasi*-3D and (c, d) 3D samples. (e) Electrical resistivity measurements followed by turning the light *on* and *off*.

Second, photoconductivity measurements were performed using a solar simulator (100 mW cm^–2^) to probe the charge transport response of the *quasi*-3D and 3D perovskites. The samples were prepared as hot-cast films, and DC electrical resistivity was measured using a four-probe method. For this experiment, the films were grown onto FTO substrates and assembled face-to-face to form FTO/perovskite/FTO devices. Data were collected in the dark and under continuous sunlight irradiation for 30 min intervals, enabling evaluation of photoconductivity response, in addition to test the device stability and charge transport relaxation processes. Figure 5e presents the time-dependent electrical resistivity measurements, with the yellow-shaded region indicating the illumination period. As expected, the electrical resistivity magnitude of the 3D sample is lower than that of *quasi*-3D due to its comparatively higher octahedral connectivity and bulk-like transport pathways. Notably, however, both systems exhibit similar photoconductive responses characterized by a rapid decrease in resistivity upon illumination. After turning the light on, the resistivity drops to approximately 10 and 12% of the corresponding dark values for the 3D and *quasi*-3D devices, respectively. Additionally, there is also a large similarity in the response and recovery times, indicating similar charge generation and recombination dynamics under these measurement conditions. The main difference emerges at longer illumination times, where the 3D does not reach steady state but instead exhibits prolonged transient behavior, with the resistivity initially decreasing and slowly increasing. Consistently, the 3D MAPbI_3_ device (Figure 5e, black curve) presents a sharp resistivity decrease under illumination, followed by recovery once the light is switched *off*.^44^ Most importantly, the *quasi*-3D perovskites display a highly comparable *on/off* photoconductivity response, demonstrating efficient collection of photogenerated charge carriers by the electrodes. These results indicate that the *quasi*-3D compound preserves a robust photoconductivity effect comparable to that of the 3D perovskite while simultaneously exhibiting enhanced stability under prolonged illumination.

Revisiting the electronic structure discussion of the *quasi*-3D samples, we first highlight the relevance of quantum and dielectric confinements in the thick-layer (*n* = 7) octahedral-sheet compound. Figure 2 indicates a red shift in the PL peak as the number of layers increases due to the weakening of the dielectric confinement. For example, for *n* = 1 and 2, the peak energies are 2.44 and 2.13 eV, respectively, as observed in the literature.^7^ The *n* = 7 sample shows a peak at 1.66 eV, closely resembling that of the 3D sample (1.61 eV), indicating a minimal dielectric confinement effect. Consequently, the band gap energies should be similar for both samples. Overall, the comparison highlights a clear stability-transport contrast between 3D MAPbI_3_ and the *quasi*-3D Ruddlesden–Popper *n* = 7 phase. While MAPbI_3_ brings about strong photoresponse, it is intrinsically more susceptible to environmental and stress-driven degradation (e.g., ion migration under heat/light/bias and moisture sensitivity), which is widely recognized as a key origin of performance loss and operational instability in 3D perovskites.^45^ Consistently, experiments in MAPbI_3_ show rapid pressure-induced PL quenching at relatively low pressure and incomplete recovery after decompression, consistent with a mechanically lattice defects and irreversible disorder. In contrast, the *quasi*-3D *n* = 7 resists substantially higher pressure before severe PL suppression and exhibits reversible PL restoration upon release, indicating an enhanced structural robustness. The *quasi*-3D sample preserves a 3D-like electrode-collected photoconductivity response under solar simulator cycling that is comparable to MAPbI_3_, while simultaneously showing markedly improved surface water resistance (higher contact angle and preserved hydrophobicity after water exposure). This outcome agrees that bulky organic spacers in RP/*quasi*-2D systems generally increase moisture/operational stability via hydrophobic, diffusion-blocking interfaces but can penalize charge transport at low *n*.^46^ However, as *n* becomes large (*quasi*-3D regime), carrier transport trends back toward 3D-like behavior.

## Conclusion

In summary, we have synthesized a series of 2D Ruddlesden–Popper (RP) samples changing the number of layers (*n*) (octahedral sheets) spaced by organic molecules and its 3D counterpart MAPbI_3_. We demonstrated that thick-layer RP perovskites in the *quasi*-3D regime (*n* = 7) can preserve 3D-like optoelectronic functionality while substantially improving resilience to external stressors. *In situ* hydrostatic-pressure PL shows a clear divergence between the two lattices: 3D MAPbI_3_ exhibits near-total PL quenching above ∼3–4 GPa and only minimal recovery after decompression, whereas the *quasi*-3D (*n* = 7) crystals sustain emission to much higher pressures (severe quenching only near ∼8 GPa) and recover their excitonic emission upon pressure release, consistent with a more stable structure. These observations align with broader understanding that 3D halide perovskites are highly susceptible to stress- and environment-assisted degradation pathways (including defect formation and ion migration), motivating stabilization strategies beyond purely 3D lattices. Importantly, this increased robustness of the *quasi*-3D phase is achieved without compromising its functional photoresponse. Wetting tests show that introducing only a minority fraction of organic spacer is sufficient to impart a markedly more hydrophobic and water-resistant surface than MAPbI_3_, while photoconductivity measurements under solar-simulator cycling reveal a comparable light-induced resistivity drop and recovery for *quasi*-3D and 3D films, indicating efficient electrode-collected photocarriers in the *n* = 7 compound alongside greater stability. These results support the central premise from the 2D layered-perovskite: low-*n* RP phases maximize moisture/thermal robustness but can impede transport, whereas the high-*n* (*quasi*-3D) limit can recover 3D-like carrier behavior while retaining some of the stability advantages associated with spacer-engineered architectures. Looking ahead, *quasi*-3D materials are therefore promising either as stand-alone absorbers or as stabilizing components in mixed-dimensional (2*D*/3D) device stacks; systematic long-term operation tests (humidity/thermal/light bias), coupled with direct probes of ion migration and interfacial energetics, should clarify how far these mechanically and chemically robust photoconductors can be pushed toward durable, high-efficiency optoelectronic devices.

## Supplementary Material



## Notes

XRD evolution across *n* (1, 2, 7, 3D), indicating structural transition; Normalized absorbance spectra at room temperature with gradual bandgap reduction toward *quasi*-3*D*/3D behavior; lattice parameters and unit cell expansion with increasing n Lattice parameters (*a, b, c*) for *n*-dependent structures: structural trend from 2D to 3D limit (PDF)
