# Supplementary material for: 2D Ruddlesden–Popper Perovskites with a Thick Octahedral Layer (n = 7) as a Robust Alternative for Energy-Related Application
Source: ACS Phys Chem Au. 2026 May 8;6(4):763–72. doi: 10.1021/acsphyschemau.6c00021 (PMC13397439; doi:10.1021/acsphyschemau.6c00021)
Supplement: Supplementary file 1 [file pg6c00021_si_001.pdf]

Supporting Information

2D Ruddlesden-Popper perovskites with thick octahedral layer (*n* = 7) as robust alternative for energy-related application

Aryane Tofanello[1], André L. M. Freitas[1], Fabio Abud[1], Leonardo Quintero[1], Ulisses F. Kaneko[2,3], Ricardo D. Reis[2], Jose A. Souza*[1]*

[1] Center for Natural and Human Sciences (CCNH), Federal University of ABC (UFABC), Santo André, São Paulo, 09210-580, Brazil

[2] Brazilian Synchrotron Light Laboratory (LNLS), Brazilian Center for Research in Energy and Materials (CNPEM), Campinas, São Paulo, 13083-100, Brazil

[3] São Paulo State University - UNESP, Institute of Geosciences and Exact Sciences (IGCE), Physics Department, Rio Claro, São Paulo, 13506-900, Brazil

Correspondent authors: aryane.tofanello@ufabc.edu.br and joseantonio.souza@ufabc.edu.br

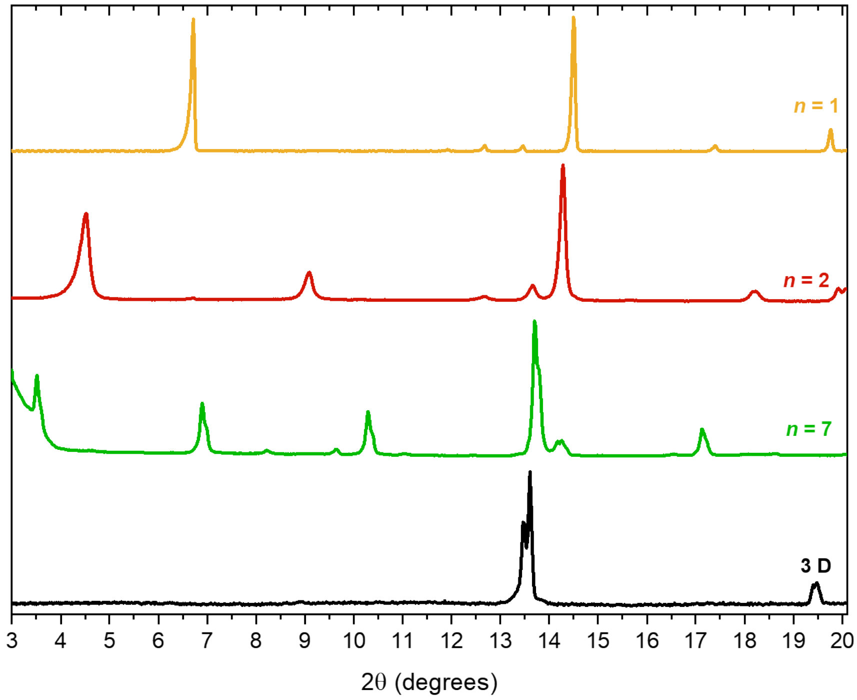

Figure S1. X-ray diffraction patterns presenting the evolution of the diffraction within the $BA_2MA_{n-1}Pb_nI_{3n+1}$ members (*n* = 1, 2, 7 and 3D) as a function of *n*.

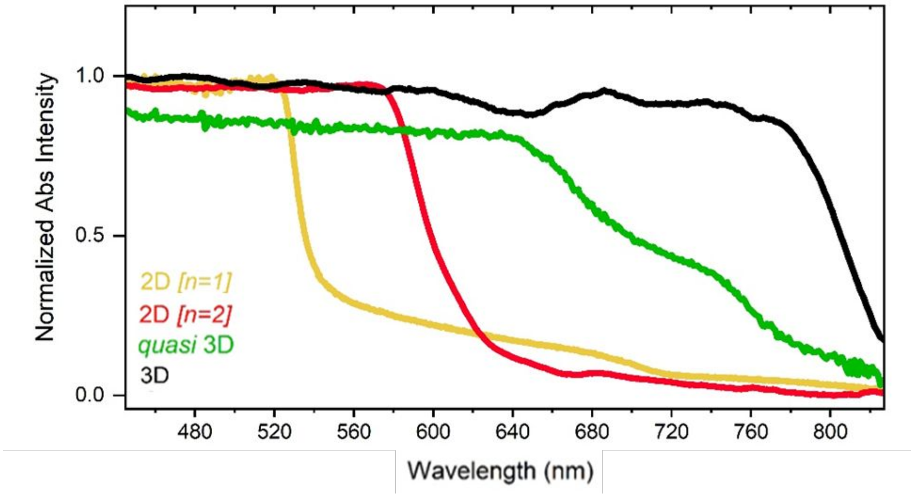

Figure S2. Normalized absorbance spectra of 2D perovskite with *n* = 1 (yellow curve), *n* = 2 (red curve), *n* = 7 - *quasi*-3D (green curve) and 3D perovskite (black curve) at room temperature.

Table 1. Lattice parameters (*a,b,c*) and unit cell volume (V) related to the *n* evolution hybrid perovskite.

n	**a(Å)**	**b(Å)**	**c(Å)**	**Volume (Å^3)**
**1**	8.84	27.34	8.98	2172.04
**2**	8.92	38.51	8.85	3039.6
**7**	8.89	101.95	8.91	8087.51
**3D (∞)**	8. 84	12.89	8.84	1007.5
